# Supplementary material for: Mapping individual differences in intermodal coupling in neurodevelopment
Source: Imaging Neurosci (Camb). 2025 Sep 22;3:IMAG.a.156. doi: 10.1162/IMAG.a.156 (PMC12455055; doi:10.1162/IMAG.a.156)
Supplement: Supplementary Material [file IMAG.a.156_supp.pdf]

# Supplementary material for “Mapping Individual Differences in Intermodal Coupling in Neurodevelopment”

## I Revisiting IMCo as a special case of CEIDR

The subject-level intermodal coupling estimated by IMCo using locally weighted correlation, denoted by $\phi_i(v)$ here, is

$$\phi_i(v) = \frac{\sum_{v^\star=1}^{V} w_{v,v^\star} \times (y_{1i}(v^\star) - \tilde{y}_{1i}(v)) \cdot (y_{2i}(v^\star) - \tilde{y}_{2i}(v))}{\sqrt{\sum_{v^\star=1}^{V} w_{v,v^\star} \times (y_{1i}(v^\star) - \tilde{y}_{1i}(v))^2} \times \sqrt{\sum_{v^\star=1}^{V} w_{v,v^\star} \times (y_{2i}(v^\star) - \tilde{y}_{2i}(v))^2}}, \tag{S1}$$

where $\tilde{y}_{1i}(v)$ and $\tilde{y}_{2i}(v)$ are local weighted means, computed by

$$\tilde{y}_{mi}(v) = \frac{\sum_{v^\star=1}^{V} w_{v,v^\star} \times y_{mi}(v^\star)}{\sum_{v^\star=1}^{V} w_{v,v^\star}},$$

and $w_{v,v^\star}$ is the weight determined by the distance $d_{v,v^\star}$ and prespecified FWHM as described in Section 2.5.1 of the paper. To build methodological connection, we consider a special case of $w_{v,v^\star}$ that

$$w_{v,v^\star} = \begin{cases} 1 & \text{if } v^\star \in N_r(v) \\ 0 & \text{otherwise.} \end{cases} \tag{S2}$$

Then, $\tilde{y}_{mi}(v) = \bar{y}_{mi}(v)$ (sample mean of $y_{mi}(v)$ within $N_r(v)$) defined in Section 2.4 (I.2) of the paper and Equation (S1) can be re-written as

$$\begin{aligned}
\phi_i(v) &= \frac{\sum_{v^\star=1}^{V} w_{v,v^\star} \times (y_{1i}(v^\star) - \bar{y}_{1i}(v)) \cdot (y_{2i}(v^\star) - \bar{y}_{2i}(v))}{\sqrt{\sum_{v^\star=1}^{V} w_{v,v^\star} \times (y_{1i}(v^\star) - \bar{y}_{1i}(v))^2}\sqrt{\sum_{v^\star=1}^{V} w_{v,v^\star} \times (y_{2i}(v^\star) - \bar{y}_{2i}(v))^2}} \\
&= \sum_{v^\star=1}^{V} w_{v,v^\star} \times \frac{y_{1i}(v^\star) - \bar{y}_{1i}(v)}{\sqrt{\sum_{v^\star=1}^{V} w_{v,v^\star} \times (y_{1i}(v^\star) - \bar{y}_{1i}(v))^2}} \times \frac{y_{2i}(v^\star) - \bar{y}_{2i}(v)}{\sqrt{\sum_{v^\star=1}^{V} w_{v,v^\star} \times (y_{2i}(v^\star) - \bar{y}_{2i}(v))^2}} \\
&= \sum_{v^\star=1}^{V} w_{v,v^\star} \times \frac{y_{1i}(v^\star) - \bar{y}_{1i}(v)}{\sqrt{\sum_{v^\star \in N_r(v)} (y_{1i}(v^\star) - \bar{y}_{1i}(v))^2}} \times \frac{y_{2i}(v^\star) - \bar{y}_{2i}(v)}{\sqrt{\sum_{v^\star \in N_r(v)} (y_{2i}(v^\star) - \bar{y}_{2i}(v))^2}} \\
&= \frac{1}{|N_r(v)| - 1} \times \sum_{v^\star \in N_r(v)} \hat{\epsilon}_{1i}(v^\star) \times \hat{\epsilon}_{2i}(v^\star) \\
&= \frac{1}{|N_r(v)| - 1} \times \sum_{v^\star \in N_r(v)} \hat{\rho}_i(v^\star),
\end{aligned}$$

where $\hat{\epsilon}_{1i}(v)$ and $\hat{\epsilon}_{2i}(v)$ are defined in Section 2.4 (I.2) and $\hat{\rho}_i(v)$ is defined in Section 2.4 (II). This result implies that IMCo's subject-level coupling ($\phi_i(v)$) is proportional to the sum of CEIDR's subject-level

coupling over $N_r(v)$ after applying the within-subject adjustment using vertices of $N_r(v)$.

Once $\phi_i(v)$ is obtained, IMCo uses GLM to compute $p$ value for individual differences. Suppose that we define (i) $\mathbf{Z}$ as matrix of $(1, \mathbf{z}_i)$ stacked in row across $N$ subjects (ii) $\mathbf{x}$ as a column vector of $x_i$, and (iii) $\boldsymbol{\phi}(v)$ as a column vector of $\phi_i(v)$ collected across $N$ subjects. Also, let $\mathbf{I}_N$ be a $N \times N$ identity matrix. Then the score test statistic for the association between $\phi_i(v)$ and $x_i$ adjusting for $\mathbf{z}_i$ is written as

$$\begin{aligned}
T_{\text{IMCo}}(v) &= \mathbf{x}^T(\mathbf{I}_N - \mathbf{Z}(\mathbf{Z}^T\mathbf{Z})^{-1}\mathbf{Z}^T)\boldsymbol{\phi}(v) \\
&\propto \mathbf{x}^T(\mathbf{I}_N - \mathbf{Z}(\mathbf{Z}^T\mathbf{Z})^{-1}\mathbf{Z}^T)\left(\sum_{v^\star \in N_r(v)} \hat{\boldsymbol{\rho}}(v^\star)\right) \\
&= \sum_{v^\star \in N_r(v)} \left(\mathbf{x}^T(\mathbf{I}_N - \mathbf{Z}(\mathbf{Z}^T\mathbf{Z})^{-1}\mathbf{Z}^T)\hat{\boldsymbol{\rho}}(v^\star)\right) \\
&= \sum_{v^\star \in N_r(v)} \left(\sum_{i=1}^{N} x_i \times (\hat{\rho}_i(v^\star) - \tilde{\beta}_0(v^\star) - \mathbf{z}_i\tilde{\boldsymbol{\beta}}(v^\star)\right) \\
&= \sum_{v^\star \in N_r(v)} U(v^\star) \\
&\propto T_r(v).
\end{aligned}$$

This result implies that the $p$ value of IMCo at vertex $v$ is equivalent to the fixed-radius cluster enhancement using score test statistics from CEIDR. When permutation to control FWER is used instead of FDR, Step V completes statistical inference done in CEIDR.

Altogether, our derivation demonstrates that, when specific weights $w_{v,v^\star}$ are given as Equation (S2), IMCo implemented with weighted correlation and FWER control reduces to a special case of CEIDR that (i) IMCo adjusts for means and variances in each modality by using the *within-subject adjustment* approach (Step I.2) and performs cluster enhancement with a fixed radius (Step IV).

## II Coupling maps

In our data analysis, we computed the average intermodal coupling at each vertex by averaging $\hat{\rho}_i(v)$ in Stage II across subjects. The resulting spatial map is presented in Figure S1.

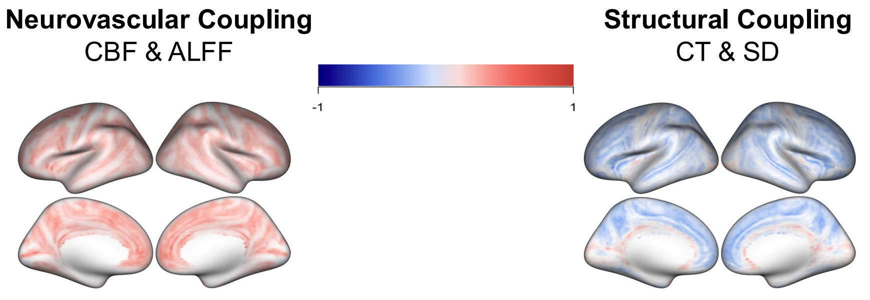


| Minimum | Q1 | Median | Q3 | Maximum |
|---|---|---|---|---|
| -0.141 | 0.073 | 0.147 | 0.215 | 0.693 |

| Minimum | Q1 | Median | Q3 | Maximum |
|---|---|---|---|---|
| -0.571 | -0.181 | -0.108 | -0.036 | 0.412 |

**Figure S1.** Average intermodal coupling map across subjects. For each vertex, the value represents the average of subject-specific coupling estimates computed in Stage II.
